# A Comparison of Proximity and Land Use Regression Traffic Exposure Models and Wheezing in Infants

**DOI:** 10.1289/ehp.9480

**Published:** 2006-10-30

**Authors:** Patrick H. Ryan, Grace K. LeMasters, Pratim Biswas, Linda Levin, Shaohua Hu, Mark Lindsey, David I. Bernstein, James Lockey, Manuel Villareal, Gurjit K. Khurana Hershey, Sergey A. Grinshpun

**Affiliations:** 1 Department of Environmental Health, University of Cincinnati Medical Center, Cincinnati, Ohio, USA; 2 Environmental Engineering Science, Washington University in St. Louis, St. Louis, Missouri, USA; 3 Department of Internal Medicine, University of Cincinnati Medical Center, Cincinnati, Ohio, USA; 4 Division of Allergy and Immunology, Cincinnati Children’s Hospital Medical Center, Cincinnati, Ohio, USA

**Keywords:** diesel, land, model, proximity, regression, spatial, traffic, use

## Abstract

**Background:**

We previously reported an association between infant wheezing and residence < 100 m from stop-and-go bus and truck traffic. The use of a proximity model, however, may lead to exposure misclassification.

**Objective:**

Results obtained from a land use regression (LUR) model of exposure to truck and bus traffic are compared with those obtained with a proximity model. The estimates derived from the LUR model were then related to infant wheezing.

**Methods:**

We derived a marker of diesel combustion—elemental carbon attributable to traffic sources (ECAT)—from ambient monitoring results of particulate matter with aerodynamic diameter < 2.5 μm. We developed a multiple regression model with ECAT as the outcome variable. Variables included in the model were locations of major roads, bus routes, truck traffic count, and elevation. Model parameter estimates were applied to estimate individual ECAT levels at infants’ homes.

**Results:**

The levels of estimated ECAT at the monitoring stations ranged from 0.20 to 1.02 μg/m^3^. A LUR model of exposure with a coefficient of determination (*R*^2^) of 0.75 was applied to infants’ homes. The mean (± SD) ambient exposure of ECAT for infants previously categorized as unexposed, exposed to stop-and-go traffic, or exposed to moving traffic was 0.32 ± 0.06, 0.42 ± 0.14, and 0.49 ± 0.14 μg/m^3^, respectively. Levels of ECAT from 0.30 to 0.90 μg/m^3^ were significantly associated with infant wheezing.

**Conclusions:**

The LUR model resulted in a range of ECAT individually derived for all infants’ homes that may reduce the exposure misclassification that can arise from a proximity model.

Many previous studies of the association between air pollution and health outcomes have relied on the distance to the pollutant source (i.e., road) as a surrogate of exposure (Ciccone et al. 1998; Hoek et al. 2002; Janssen et al. 2003; Lin et al. 2002; Ryan et al. 2005; van Vliet et al. 1997; Venn et al. 2001). An assumption of these proximity models is that nearness to the emission source is an appropriate surrogate for exposure (Jerrett et al. 2005). Although the application of proximity models is generally straightforward, this method is limited in the assumption that subjects within a given distance of an exposure source are equally exposed. Land use regression (LUR) models may be an appropriate alternative to assess intraurban air pollution caused by the large number of pollutant sources and the rapid decay of many urban pollutants (Briggs et al. 1997). This methodology has been successfully used to model nitrogen dioxide and particulate matter with aerodynamic diameter < 2.5 μm (PM_2.5_) (Brauer et al. 2003; Briggs et al. 1997, 2000; Gilbert et al. 2005; Ross et al. 2006). The advantages of this approach include the use of monitoring data and the ability to model small-area variations in pollution with the use of buffers surrounding the prediction area. The present study is the first to employ this methodology to model a marker of diesel exhaust particulates (DEP)—the sampled elemental carbon (EC) attributable to traffic sources (ECAT)—in an epidemiologic study.

Studies have shown a correlation between air pollution and allergic and respiratory diseases (Brunekreef et al. 1997; Lin et al. 2002; Venn et al. 2001). In contrast to the combustion of gasoline, the combustion of diesel fuel produces up to 100 times more particulates, and most are fine (0.1–2.5 μm) or ultrafine (< 0.1 μm) in size [U.S. Environmental Protection Agency (EPA) 2002]. Because of their small size, DEP can reach the nasal and peripheral airways on inhalation, enhancing the allergic immune response and increasing production of immunoglobulin E (Pandya et al. 2002; Riedl and Diaz-Sanchez 2005). Exposure to these particulates during infancy while the immature immune system is developing may have contributed to the increase in allergic diseases over the last two decades (Sherrill et al. 1999).

We have reported that infants in the Cincinnati Childhood Allergy and Air Pollution Study (CCAAPS) whose residences were within 100 m of stop-and-go bus and truck traffic were at an increased risk for wheezing before 1 year of age compared with infants unexposed to truck and bus traffic [adjusted odds ratio (AOR) = 2.50; 95% confidence interval (CI), 1.15–5.42] (Ryan et al. 2005). This association, however, was not evident in those infants who lived < 400 m from the nearest interstate, despite the greater average volume of trucks. The use of residential proximity to truck and bus traffic, however, is a potential limitation because isotropic dispersion is assumed and large variations in the number of trucks within given exposure proximities are likely to result in exposure misclassification. Therefore, to address the need for the development of models that are capable of assessing intraurban air pollution exposures (Brauer et al. 2003; Brunekreef and Holgate 2002; National Research Council 2002) and to improve on our prior exposure model, we report now on the application of a LUR model for ECAT, a marker of diesel combustion in the greater Cincinnati, Ohio, area. The model is applied to the identical subset of the CCAAPS cohort previously analyzed using a proximity model (Ryan et al. 2005). The purpose of this study is to develop a LUR model of estimated DEP exposure, compare the estimated exposures generated from the LUR model with exposure categories designated using the previous proximity model, and determine whether there is an association between the LUR model derived exposures to ECAT and infant wheezing before 1 year of age.

## Methods

### Cohort description

CCAAPS is an ongoing prospective birth cohort study. The study’s purpose is to determine whether infants who are exposed to DEP are at an increased risk for developing atopy and allergic respiratory diseases and to determine whether this effect is modified in a genetically at-risk population. The study methods, population, and sampling methodology are described in detail elsewhere (Hu et al. 2006; LeMasters et al. 2006; Martuzevicius et al. 2004; Ryan et al. 2005). Briefly, eligible infants were identified from birth records, and the addresses obtained using these records were geocoded using EZLocate software (TeleAtlas Inc., Menlo Park, CA). Only those infants whose address was assigned the highest match code (TeleAtlas Inc. 2006) were included in this analysis. We computed the distance to the nearest road with > 1,000 trucks daily (“major road”) using the geoprocessing extension available for ArcView GIS 3.2 (Environmental Systems Research Institute Inc., Redlands, CA). Institutional review board approval was obtained before subject recruitment, and all enrolled parents gave written informed consent before their own and their infants’ study participation. Parents were recruited when infants were approximately 6 months of age and screened for allergy symptoms. Infants of atopic parents [confirmed with a skin prick test (SPT)] were enrolled. CCAAPS has continued prospectively evaluating enrolled infants with annual SPTs, physical examinations, questionnaires, and environmental exposure assessments, including exposure to house dust, mold, and environmental tobacco smoke (ETS) (Biagini et al. 2006; Cho et al. 2006; Osborne et al. 2006).

### Proximity model of exposure

A proximity model of exposure was previously developed and applied to the CCAAPS cohort (Ryan et al. 2005). Briefly, exposure to moving traffic was determined whether an infant’s residence was within 400 m of an interstate or within 100 m of a state route with a speed limit ≥50 mph. A distance of 400 m was chosen based on our prior results that found concentrations of key elements indicative of traffic sources were elevated up to 400 m (Reponen et al. 2003). Exposure to stop-and-go traffic was determined if an infant’s residence was within 100 m of a bus route, within 100 m of a state route with a speed limit < 50 mph, or both. If an infant resided > 400 m from the nearest interstate,> 100 m from the nearest state route, and > 100 m from the nearest bus route, the infant was placed in the unexposed category.

### Ambient air sampling methodology

**Figure 1:**
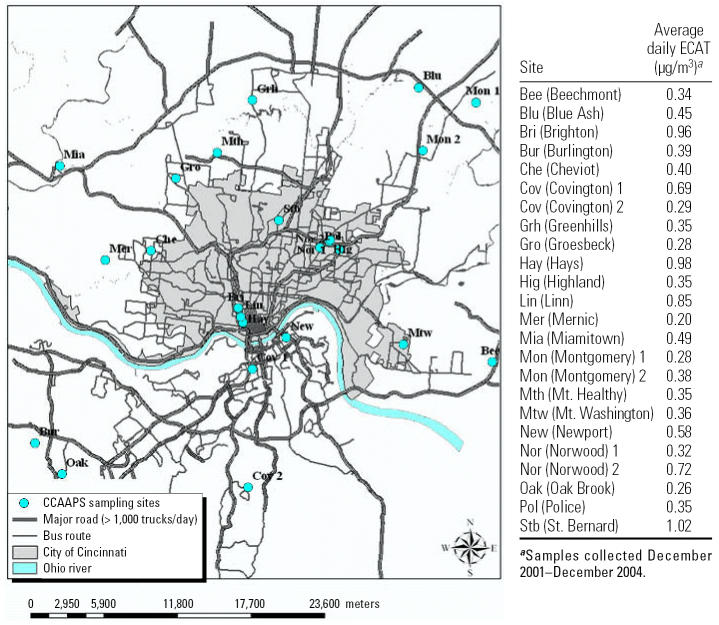
CCAAPS ambient PM_2.5_ monitoring network.

The CCAAPS ambient air sampling network contains 24 sites (Figure 1) selected on the following characteristics: city area, proximity of pollution sources including highway and state routes, location of the study population, location relative to the major interstate highway corridors, and location relative to the predominant wind direction (Martuzevicius et al. 2004). The annual measurement campaign was performed from December 2001 through December 2004. At each cycle, sampling was conducted simultaneously at four to five sites over different seasons.

PM_2.5_ samples were collected on 37-mm Teflon membrane filters (nominal pore size, 1 μm; Pall Gellman, Ann Arbor, MI) and 37-mm quartz filters (Whatman, Kent, ME) with Harvard-type Impactors (Air Diagnostics and Engineering, Harrison, ME). Standardized operating procedures for the filter media preparation, gravimetric operations, and sampling were followed (Hu et al. 2006; Martuzevicius et al. 2004). The Teflon filters were conditioned for at least 24 hr in a humidity chamber at a relative humidity of 30–40% with a temperature of 22–24°C for temperature and humidity equilibration at Washington University and weighed before and after the sampling to determine PM_2.5_ mass concentrations (Hu et al. 2006). The Teflon filters were then analyzed by X-ray fluorescence (XRF) to determine elemental concentrations (Chester Labnet, Tigard, OR) for a total of 38 elements. The quartz filters were sectioned with one-half analyzed by the thermal-optical transmittance (TOT) technique using the National Institute for Occupational Safety and Health (NIOSH)-5040 method (Birch and Cary 1996) to determine EC and organic carbon (OC) concentrations. The other half was frozen and preserved. Of the total 609 samples, 147 were collocated on Teflon and quartz filters. These 147 were analyzed by the TOT technique to determine EC concentrations. Given the importance of EC to this study, we used an alternative method, optical reflectance, to estimate the EC concentrations from the Teflon filters. The reflectance of ambient aerosols deposited on the Teflon filter was measured by a reflectometer (EEL model 43; Diffusion System Ltd., London, UK). The absorption coefficient (A_bs_) of the aerosol-loaded Teflon filters was calculated according to International Standard 9835 (ISO 1993). The absorption coefficient was correlated with EC from NIOSH-5040 thermo-optical transmittance analysis for days when quartz filter sampling was conducted. The EC concentrations in the remaining 462 samples were determined by the reflectance method. Field and laboratory blanks were routinely analyzed, with details reported elsewhere (McDonald et al. 2004).

### Estimation of DEP exposure

We first used the multivariate receptor model, UNMIX (Henry 2000, 2003; Henry et al. 1999), to determine source signatures in the airshed. We identified four different source categories, with one attributed to traffic sources. We compared the estimated source profiles with those obtained from measurements conducted for cluster sources of trucks and buses (Hu et al. 2006). These profiles provided some credence that the estimated traffic source signature in the measured region was dominated by diesel sources. We then determined the ECAT by both UNMIX and the chemical mass balance (Watson et al. 1990) model. The fraction of the total ECAT source category was calculated, and used as the marker of estimated DEP exposure. It is well known that various sources contribute to ambient levels of measured EC (Schauer 2003; White 2001). We use the fraction of the ambient EC attributed to traffic sources that is dominated by diesel exhaust. Thus, ECAT provides a greater degree of confidence in the results compared with using the entire PM_2.5_ fraction.

### LUR model

To determine the significance of site characteristics anticipated to predict average daily ECAT levels at monitoring locations, we used a regression based approach (Brauer et al. 2003; Briggs et al. 1997; Gilbert et al. 2005; Ross et al. 2006). This technique predicts the pollution concentrations at given sampled locations based on the surrounding land use and traffic characteristics and has been shown to be more effective than other interpolation methods for modeling pollutants with high variability within a small area (Briggs et al. 1997; Lebret et al. 2000). The levels of ECAT are averaged to minimize the effect of seasonal and temporal variation and to account for the different time and season during which each infant is exposed before 1 year of age. Sampled levels of ECAT are assumed to be independent because of the large spatial separation and differences among location characteristics. The land use and traffic independent variables were chosen based on previously validated LUR models (Brauer et al. 2003; Briggs et al. 1997; Gilbert et al. 2005; Ross et al. 2006) and our previous results indicating that exposure to bus routes was a significant risk factor for wheezing in infants (Ryan et al. 2005). We chose buffer distances of 100 m and 400 m for bus and major roads, respectively, on the basis of the geographic distribution of the enrolled infants and previous studies of the decay of particulates from roadways, and to enable contrasts between the proximity and LUR models with equidistant geographic locations (Reponen et al. 2003; Ryan et al. 2005; Zhu et al. 2002).

*A priori*, we determined that the geographic information to be included would be elevation, length of major roads (defined as > 1,000 trucks daily) within a 400-m buffer, length of bus routes within a 100-m buffer, total number of trucks within a 400-m buffer per day, distance to the nearest major road, distance to the nearest bus route, and land use. Elevation data were obtained using a 7.5-min digital elevation model (DEM) producing 30-by 30-m cells (United States Geological Survey 1997). Traffic count and road data were obtained from the Ohio Department of Transportation (2004) and the Kentucky Transportation Cabinet (2006). Average daily truck and car counts are provided by the Ohio Department of Transportation (2004) and Kentucky Transportation Cabinet (2006) for each road segment for the years 2001–2004. Local bus routes were obtained from regional transit authorities (D. Shuey, personal communication). Land use was determined using the National Land Cover Database (U.S. EPA 2001) and summarized as either low intensity (impervious surface < 20%), medium intensity (impervious surface 20–50%), or high intensity (impervious surface > 50%) (Homer et al. 2004). Land use and traffic characteristics were derived for each sampling location and participating infant’s birth residence using ArcGIS 9.0 (Environmental Systems Research Institute Inc.).

In the regression model, ECAT was log-transformed, given a relatively lognormal distribution of ECAT levels. All land use variables and traffic variables were considered for inclusion in a multiple linear LUR model with log-transformed ECAT as the dependent variable. Correlations among pairs of predictor variables, and associations between each predictor variable and ECAT were reviewed to assist in model building. Modification in the scale of a selected predictor variable was assessed by comparing coefficients of determination (*R*^2^) and calculation of Akaike’s information criterion (Akaike 1973). To allow the log-transformation to be applied to independent variables with zero values, a value of zero was replaced by 0.01. Influence statistics and residual plots were obtained to assess the fit of the final model. We validated the final regression model by a comparison of prediction errors obtained by simulating 1,000 jack-knifed data sets. For each data set, parameter estimates estimated from the LUR model for ECAT were obtained on a subset of 19 locations, and were applied to the excluded locations to predict ECAT. We compared the averages of the 1,000 mean-squared errors of the model-based and test data sets to assess the applicability of the final model to other locations. ECAT levels were subsequently estimated for the homes of all enrolled infants using the parameter estimates obtained from the LUR model and were compared with the exposure indices resulting from the proximity model.

### Health outcome data

One study objective is to compare the previously reported association between exposure to stop-and-go traffic and wheezing in infants and the association between exposure level derived from the LUR model and the identical outcome. Therefore, wheezing without a cold was designated if the parent reported their infant wheezing without a cold at approximately 6–7 months of age and at least one other occasion on a monthly diary before the infant’s first birthday (Ryan et al. 2005).

### Statistical analyses

We compared the findings from the proximity model categories of exposure and the estimates of ECAT levels derived from the LUR model. We computed histograms of ECAT levels for infants previously designated as unexposed, exposed to stop-and-go traffic, or exposed to moving traffic using S-Plus (version 4.5 Professional; Insightful Corporation, Seattle, WA). We compared the mean and median levels of ECAT derived for each previous exposure category using *t*-tests and the Wilcoxon rank-sum test as appropriate, using SAS (version 9.1 for Windows; SAS Institute Inc, Cary NC). We examined the association between ECAT exposure and wheezing without a cold using conditional logistic regression. Adjustment is made for maternal smoking (maternal self-report of smoking), child care attendance, sex, race (white/minority), breast-feeding (maternal report of breast-feeding < 1 week, 1–4 weeks, > 4 weeks), pet ownership, and report of visible mold in the home by a study inspector.

## Results

In total, 622 infants fulfilled the eligibility requirements at average age 7.5 months (± 2.4 months), and 8.0% (*n* = 50) were reported to have recrurrent wheezing without a cold (Ryan et al. 2005). Infants were previously categorized as unexposed (*n* = 347), exposed to stop-and-go truck and bus traffic (*n* = 99), or exposed to moving truck and bus traffic (*n* = 176).

A summary of the mean, minimum, and maximum values of land use characteristics is shown in Table 1 for sampling sites and the residence of the infants at birth. In general, mean values for the sites and children’s homes were similar with regard to elevation, average daily truck count, and length of bus routes. Home residences, however, were approximately three times more distant from major roads and twice as far from the nearest bus route than were the sampling sites. As shown in Figure 1, the average ECAT levels calculated for each sampling site showed a 5-fold difference in levels and ranged from 0.20 to 1.02 μg/m^3^.

All land-use and traffic variables were considered first for inclusion in a simple linear LUR model, with log-transformed ECAT as the dependent variable (Table 2). Variables having a relatively low correlation with other predictor variables and a significant association with ECAT (*p* < 0.10) were selected for inclusion in the final multiple linear regression model (Table 2). Results obtained from the jackknifed data sets comparing prediction errors from 1,000 simulations on random subsets of 19 sampling sites showed that the estimated parameters from the LUR model for ECAT were robust. The final model *R* ^2^ was 0.75 and included elevation, number of trucks within 400 m of the sampling site, and the length of bus routes within 100 m of the sampling site. Elevation was the most significantly associated variable and was inversely associated with ECAT.

**Figure 2:**
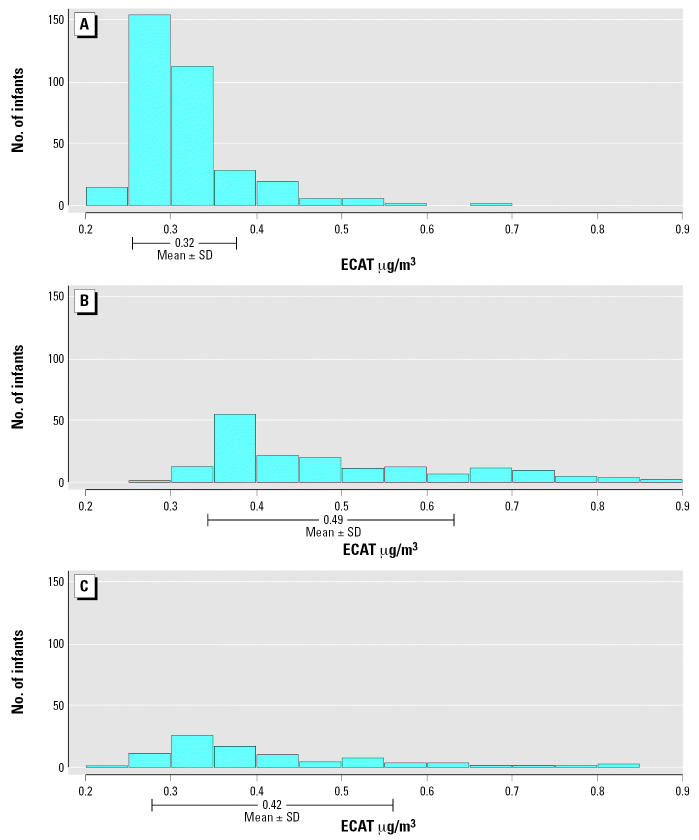
Histograms with mean ± SD of LUR-derived ECAT for infants previously categorized as (*A*) unexposed to traffic, (*B*) exposed to moving traffic, and (*C*) exposed to stop-and-go traffic.

After the development of the LUR model, 100- and 400-m buffers were created surrounding each infant’s residence. We calculated the elevation, average daily truck count on major roads within 400 m, and the length of bus routes within 100 m for each infant’s residence. An estimate of the level of ECAT at each infant’s residence was derived using the parameter estimates obtained from the LUR model. ECAT was derived only for the infant’s residence because only 14.4% of infants were reported to attend child care. Summary statistics for the sampled ECAT levels and estimated ECAT levels are presented in Table 3. In general the mean, median, SD, and range were similar for the LUR-derived ECAT at the infants’ homes compared with the monitored ECAT levels. Figure 2 displays the distribution of LUR-derived ECAT for each previously designated exposure category. The mean (± SD) of the ECAT estimates for infants previously categorized as unexposed, exposed to stop-and-go traffic, or exposed to moving traffic are 0.32 ± 0.06 μg/m^3^, 0.42 ± 0.14 μg/m^3^, and 0.49 ± 0.14 μg/m^3^, respectively (Table 3). Both the stop-and-go and moving categories overlap within 1 SD of the “unexposed” category. For example, among infants designated as “unexposed” to truck and bus traffic in the proximity model, the range of ECAT is 0.23–0.68 μg/m^3^. The ranges of ECAT among infants previously designated as exposed to stop-and-go or moving traffic were 0.25–0.83 μg/m^3^ and 0.29–0.88 μg/m^3^, respectively. The Wilcoxon rank-sum test showed the median values of ECAT derived for infants categorized as exposed to stop-and-go traffic (0.37 μg/m^3^) and moving traffic (0.44 μg/m^3^) were each significantly higher (*p* < 0.05) than the median value of ECAT for infants categorized as unexposed (0.30 μg/m^3^).

AORs for the association between the LUR-derived ECAT and wheezing without a cold are presented in Table 4. The lowest estimated exposure to ECAT among infants was approximately 0.2 μg/m^3^ (Table 3) and was set as the reference level. The association between ECAT and wheezing without a cold remains significant after adjustment for maternal smoking, child care attendance, sex, race, breast-feeding, pet ownership, and visible mold. At 0.5 μg/m^3^ there is almost a 2-fold increase in wheezing (AOR = 1.86; 95% CI, 1.02–3.39) and at 0.9 μg/m^3^ it increases to > 4-fold (AOR = 4.26; 95% CI, 1.06–17.26).

## Discussion

In general, the resulting LUR model for ECAT was comparable to those previously developed for other urban areas with regard to the explained variability (*R*^2^) and the geographic variables used (Brauer et al. 2003; Briggs et al. 1997, 2000; Gilbert et al. 2005; Ross et al. 2006). The distribution of ECAT within each of the three originally designated exposure categories showed a range of ECAT exposure levels. The range of LUR-derived ECAT exposure within proximity exposure category overlapped one or both of the other two categories (Figure 2). Hence, the observed variability of LUR-derived ECAT exposure is an advantage of the LUR model.

The mean value of ECAT among infants previously designated as unexposed (0.32 μg/m^3^) was lower than the mean values of ECAT among infants previously designated as exposed to stop-and-go traffic (0.42 μg/m^3^) and moving traffic (0.49 μg/m^3^). This difference supports confidence in the model. The similar ranges of ECAT values among infants previously designated as exposed to stop-and-go and moving traffic support the hypothesis that actual exposure to DEP depends not only on the amount of traffic but also on the elevation of the residence in relation to traffic, the intensity or number of trucks, and the proximity to particular types of traffic (i.e. bus routes), which also represents considerable stop-and-go movement.

The final LUR model contained elevation, average daily number of trucks within 400 m, and the length of bus routes within 400 m, and accounts for > 75% of the variability in the sampled levels of ECAT. Of these, elevation was the most significant independent variable (Table 2). The greater Cincinnati area lies within the Ohio and Kentucky River Valley Region and is commonly referred to as the “City of Seven Hills.” The topography of this area is notable for the valleys surrounding four sizeable rivers that converge in the region. In addition, five interstates converge, I-71, I-74, I-275, I-471, and I-75, the latter of which is constructed within the Mill Creek Valley, contributing to > 60,000 trucks passing through the region daily. This combination of topography and traffic has contributed to frequent U.S. EPA nonattainment ratings. The finding that topography plays a critical role in the level of ECAT exposure in the Ohio River valley emphasizes the importance of including local geographic variables in land-use regression models. For example, Ross et al. (2006) report that the distance to the Pacific Ocean is a significant predictor for NO_2_ levels in San Diego County, California. LUR models allow adaptation to varying locations, and therefore relevant local geographic and land-use variables should be included (Jerrett et al. 2005).

In the present study we compared the results of proximity and LUR models of exposure to truck and bus traffic. In addition, we examined the association between LUR derived exposure to ECAT and wheezing in infants. ECAT is significantly associated with wheezing in infants supporting our previous finding of a significant association with stop-and-go traffic exposure. Furthermore, AORs were calculated for incremental increases in ECAT levels within the observed sampled range of ECAT values. Our previous study found that infants exposed to stop-and-go traffic had an AOR of 2.5 (95% CI, 1.15–5.42) (Ryan et al. 2005). This reported odds ratio is comparable to a 0.4- to 0.5-μg/m^3^ increased level of ECAT exposure—roughly the mean levels of ECAT for the infants originally designated as exposed to moving or stop-and-go traffic. The lack of association between exposure to moving traffic and wheezing previously reported (Ryan et al. 2005) may be a result of the wide range of ECAT exposures within the proximity model–designated exposure categories. As shown in Figure 2 and Table 3, the median exposure to ECAT among infants designated as being exposed to moving traffic is 0.44 μg/m^3^. In contrast, approximately 95% of the infants previously designated as unexposed are estimated to have ECAT at or below this level.

The issue remains, however, that moving traffic was not associated with wheezing, although the mean value of ECAT was higher in the moving category than in the stop-and-go category. This discrepancy may be explained by the distribution of infant residences within LUR buffers, LUR model parameters, or the housing stock in the moving and stop-and-go categories. Although 100- and 400-m buffers were used to determine exposure in the proximity models and served as buffers to represent exposures in the LUR model, the median distances to the nearest stop-and-go and moving traffic are 43 and 252 m, respectively. In addition, elevation, the most significant parameter in the LUR model, varies according to the previous exposure categories. The mean (± SD) elevation for infants previously categorized as unexposed, exposed to stop-and-go, and exposed to moving traffic are 812.8 ± 84.6, 767.9 ± 123.2, and 741.6 ± 123.8 m, respectively. The housing stock also may vary significantly among infants categorized as exposed to stop-and-go and moving traffic. We previously reported that infants categorized as exposed to stop-and-go traffic are more likely to have parents with an annual household income < $40,000 than infants exposed to moving traffic (Ryan et al. 2005). The housing stock and use of air conditioning helping to minimize indoor exposures may differ among these exposure categorizations. Therefore, although the LUR-derived estimates of ECAT exposure are higher for the moving category, the actual indoor exposure of ECAT may be higher among infants categorized as exposed to stop-and-go traffic because of proximity to roads and housing. In addition, previous studies have found the level of diesel-related emissions to be even higher in city streets affected by heavy truck and bus traffic (Lena et al. 2002). Thus, the location of our sampling sites primarily near interstates and the resulting model may underestimate the level of DEP exposure in infants previously categorized as exposed to stop-and-go traffic.

A possible limitation of this study, however, is the specificity of ECAT as a marker of truck traffic and/or diesel emissions. Our marker, ECAT, is more robust than ambient EC as a marker of DEP because it is the calculated EC fraction from all traffic sources. This portion of EC averaged over various seasons at each sampling site has resulted in levels of ECAT lower than those reported for ambient EC sampling alone in an urban area (Lena et al. 2002). Nevertheless, to address the specificity of ECAT as a marker of DEP, our future plans include the use of temperature-resolved carbon analysis by the IMPROVE method, which can delineate subfractions of EC and OC (Chow et al. 1993). This technique will further describe the separate contributions to ambient PM_2.5_ of diesel and gasoline combustion and better characterize the contribution of diesel exhaust particles (Chow et al. 2004; Kim and Hopke 2005). Further, average daily truck count was significantly associated with sampled ECAT levels and was selected to be an independent variable in the final LUR model. This average truck count, however, is significantly correlated (*R*^2^ = 0.44, *p* < 0.01) with the average daily car count. In addition, the residential address used to assess traffic exposure in both models was derived from the address reported by the parent at the time of the initial questionnaire administration. It is possible, therefore, that a small number of infants may have changed residential locations between the time of parental interview (infant age 7.5 ± 2.4 months) and the infant’s first examination (age 13.6 ± 2.6 months). As the cohort ages, residential locations will be assessed and exposure estimates derived based on changes in location.

In conclusion, we have applied a land-use regression model to estimate infants’ exposure to ECAT and have compared the resulting estimates to exposures determined by a proximity model. Infants previously categorized as unexposed had the lowest mean value of ECAT when compared with infants previously categorized as exposed to either moving or stop-and-go traffic. The range of ECAT estimates within formerly designated discrete exposure categories, however, demonstrated one limitation of a proximity model. We have also demonstrated an association between ECAT exposure and wheezing. Wheezing during infancy was based on parental report, however, and may not be predictive of future development of asthma. The CCAAPS cohort will be followed and evaluated throughout childhood with more objective measures of airway inflammation and/or obstruction. The finding of a potential association between ECAT and respiratory health effects may lead to public health interventions including vehicle emission standards and determining appropriate distances from major roadways for building homes and schools. In summary, we have concluded that an infant’s geographic location within an urban area may highly influence the level of air pollutant exposure and resulting health effects and may be particularly potent in susceptible infant populations.

## Figures and Tables

**Table 1:** Summary statistics for land-use variables at sampling sites and infants' homes.

Variable	Mean ± SD	Minimum	Median	Maximum
Sampling sites (*n* = 24)				
Elevation (m)	222 ± 46	152	238	281
Average daily truck count on major roads within 400 m^a^	5,858 ± 10,034	0	302	43,214
Length (m) of bus routes within 100 m^a^	57 ± 89	0	0	254
Length (m) of major roads within 400 m^a^	411 ± 473	0	415	2,025
Distance to the nearest major road (m)	558 ± 605	26	334	2,955
Distance to the nearest bus route (m)	852 ± 1,698	7	234	7,967
Land use designation^b^	*—*	*—*	*—*	*—*
Infants' homes (*n* = 622)				
Elevation (m)	239 ± 33	149	248	294
Average daily truck count on major roads within 400 m^a^	4,077 ± 8,556	0	0	63,720
Length (m) of bus routes within 100 m^a^	35 ± 82	0	0	640
Length (m) of major roads within 400 m^a^	217 ± 320	0	0	1,452
Distance to the nearest major road (m)	1,668 ± 1,980	< 1	356	8,728
Distance to the nearest bus route (m)	1,516 ± 2,621	< 1	368	25,335
Land use designation^c^	*—*	*—*	*—*	*—*

a Includes sampling sites where no bus routes/major roads are located within 400-m buffer.

b Low intensity = 59%, medium intensity = 25%, high intensity = 16%; summary statistics not available.

c Low intensity = 73%, medium intensity = 24%, high intensity = 3%; summary statistics not available.

**Table 2:** Results of linear regression models for prediction of ECAT at sampling locations.

Variable	β	*p*-Value	Model *R*^2^
Simple linear regression model results			
Elevation (m)^a^	−1.07	< 0.01	0.66
Average daily truck count on major roads within 400 m^a^	0.07	< 0.01	0.29
Length (m) of bus routes within 100 m^a^	0.09	0.07	0.14
Length (m) of major roads within 400 m	0.07	0.11	0.11
Distance to the nearest major road (m)	−0.08	0.28	0.05
Land use	0.02	0.39	0.03
Distance to the nearest bus route (m)	−0.01	0.74	0.01
Multiple linear regression model results			
Intercept	0.34	< 0.01	
Elevation (m)	−0.85	< 0.01	0.75
Average daily truck count on major roads within 400 m^b^	0.04	0.03	
Length (m) of bus routes within 100 m	0.04	0.14	

a Included in the final multiple linear regression model (*p* < 0.10).

b Trucks/day/1,000.

**Table 3:** Summary statistics for sampled and model-derived average daily ECAT levels (μg/m^3^).

Variable	Mean ± SD	Minimum	Median	Maximum
Sampled locations (*n* = 24)	0.49 ± 0.25	0.20	0.37	1.02
All infants’ homes (*n* = 622)	0.38 ± 0.13	0.23	0.34	0.88
Infants unexposed in proximity model (*n* = 347)	0.32 ± 0.06	0.23	0.30	0.68
Infants exposed to stop/go traffic in proximity model (*n* = 99)	0.42 ± 0.14	0.25	0.37	0.83
Infants exposed to moving traffic in proximity model (*n* = 176)	0.49 ± 0.14	0.29	0.44	0.88

**Table 4:** AORs^a^ (95% CIs) for ECAT exposure levels and wheezing without a cold.

Exposure to ECAT (μg/m^3^)	AOR (95% CI)
0.2	1.00 (reference)
0.3	1.23 (1.01–1.50)
0.4	1.51 (1.01–2.26)
0.5	1.86 (1.02–3.39)
0.6	2.29 (1.03–5.09)
0.7	2.82 (1.04–7.65)
0.8	3.46 (1.05–11.49)
0.9	4.26 (1.06–17.26)

a Adjusted for sex, race, maternal smoking, child care attendance, breast-feeding, pet ownership, and visible mold in the home.
